# Trigeminal neuralgia associated with cerebellar pial arteriovenous fistula: A case report

**DOI:** 10.1097/MD.0000000000018873

**Published:** 2020-01-17

**Authors:** Shenghu Wang, Jun Mo, Shiying Gai, Changjiang Ou, Yili Chen

**Affiliations:** Department of Neurosurgery, Fourth Affiliated Hospital, School of Medicine, Zhejiang University, Yiwu, Zhejiang, China.

**Keywords:** cerebellar pial arteriovenous fistula, endovascular embolization, trigeminal neuralgia

## Abstract

**Rationale:**

Trigeminal neuralgia (TN) is frequently associated with compression at the root entry zone of the trigeminal nerve by an aberrant loop of an artery, tributaries of the petrosal vein, tumors, aneurysm, and vascular malformation. TN associated with a cerebellar pial arteriovenous fistula (PAVF) has not been described previously.

**Patient concerns:**

A 65-year-old man presented with right-sided TN. Cerebral angiography revealed a right cerebellar PAVF and magnetic resonance imaging demonstrated a mixed compression of the petrous vein complex and anterior inferior cerebellar artery at the right trigeminal nerve.

**Diagnosis:**

Due to the patient's symptoms, radiographic findings, he was diagnosed with TN and PAVF.

**Interventions:**

Coiling combined with use of the liquid embolic agent Onyx was used for the complete embolization of the fistula.

**Outcomes:**

Complete relief of the pain was achieved 3 months after endovascular treatment, and the patient has remained pain-free during 2 years of follow-up.

**Conclusions:**

Endovascular treatment with a combination of coils and Onyx embolization is an effective approach for complete resolution of rarely occurring TN caused by mixed venous and arterial compressions associated with cerebellar PAVF.

## 1 Introduction

Trigeminal neuralgia (TN) is frequently associated with compression at the root entry zone (REZ) of the trigeminal nerve by an aberrant loop of artery,^[1]^ tributaries of petrosal vein,^[2]^ tumors,^[3]^ aneurysms,^[4]^ and vascular malformation.^[5,6]^ However, TN associated with a cerebellar pial arteriovenous fistula (PAVF) has not been described previously. In this case study, we report a unique case of TN caused by a mixed compression of petrosal venous complex and anterior inferior cerebellar artery (AICA) associated with a cerebellar PAVF. Complete occlusion of the fistula with coils and Onyx embolization resulted in gradual elimination of facial pain. Patient has provided informed consent for publication of the case.

## 2 Case presentation

**Figure 1:**
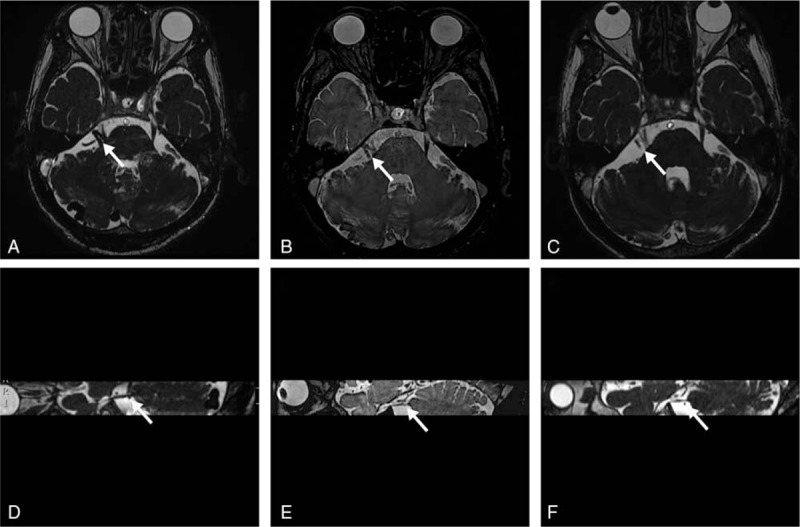
(A) Preoperative axis Fiesta sequence showed the enlarged petrosal venous complex (white arrow) compressed the right trigeminal nerve root. (B) Axis Fiesta sequence showed shrinkage of the right petrosal venous complex (white arrow) at 1 week after operation. (C) Axis Fiesta sequence showed that the right petrosal venous complex had shrunk again. (D) Preoperative sagittal Fiesta sequence showed that the ACIA (white arrow) compressed the root of trigeminal nerve. (E) Sagittal Fiesta sequence showed that the ACIA (white arrow) had shrunk at 1 week after operation. (F) Sagittal Fiesta sequence showed that the ACIA (white arrow) had shifted from the trigeminal nerve at 6 months after operation. AICA = anterior inferior cerebellar artery.

**Figure 2:**
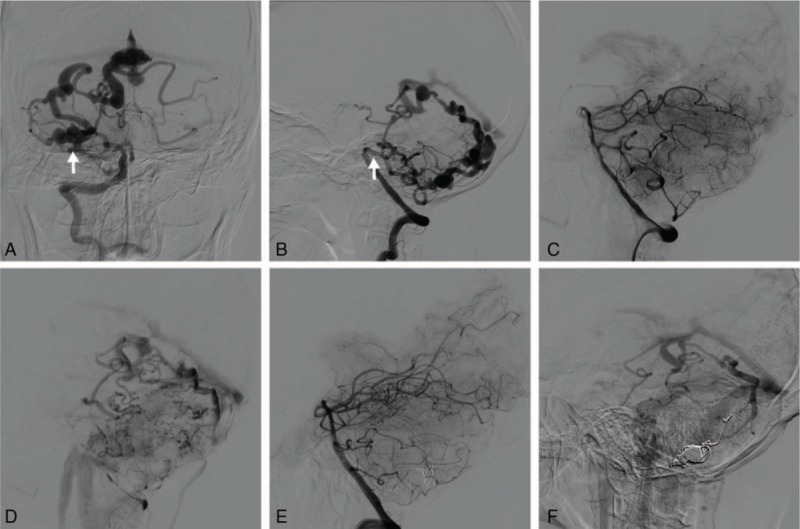
(A) Preoperative right anteroposterior vertebrobasilar angiography showed a cerebellar PAVF (white arrow) with enlarged draining veins to Galen vein. (B) Preoperative right lateral vertebrobasilar angiography showed that the PAVF was fed by the PICA, and both PICA and AICA originated from a common trunk (white arrow). (C) Arterial phase of right lateral vertebrobasilar angiography after the operation. (D) Venous phase of right lateral vertebrobasilar angiography after the operation. (E) Normal appearance of arterial phase of right lateral vertebrobasilar angiography 6 months after the operation. (D) Venous phase of right lateral vertebrobasilar angiography 6 months after the operation showed no venous anomaly. AICA = anterior inferior cerebellar artery, PAVF = pial arteriovenous fistula, PICA = posterior inferior cerebellar artery.

A 65-year-old male patient was referred to our department with a chief complaint of shock-like, stabbing pain involving the right side of the lower face for the past 2 years. He experienced a lancinating pain lasting for several minutes, which was induced by chewing, swallowing, brushing his teeth, and even talking. He revealed that the pain was aggravated when he was lying down to sleep. The pain was initially treated with carbamazepine (600 mg/d), and the patient was relieved of symptoms for a period of 6 months. However, he started to experience the pain again with increasing frequency despite high-dose carbamazepine. No exceptional medical history was reported by the patient. Neurological examination on admission was unremarkable. Three-dimensional fast imaging employing steady-state acquisition (3D FIESTA) magnetic resonance imaging (MRI) revealed a right cerebellar vascular lesion with an enlarged petrosal venous complex. Compressions at the REZ of the right trigeminal nerve by the dilated petrosal venous complex and AICA were also detected (Fig. 1A and D). Additional digital subtraction angiography (DSA) demonstrated a high-flow cerebellar PAVF fed by the posterior inferior cerebellar artery (PICA), with retrograde venous drainage via cerebellar cortical veins and the Galen vein to the petrosal vein (Fig. 2A and B).

Endovascular treatment was carried out under general anesthesia. Endovascular access via the right common femoral artery puncture was achieved, and a 6-Fr guiding catheter was placed in the left vertebral artery. Selective catheterization of the PICA was performed using an Echelon 10 microcatheter (Covidien/ev3, Irvine, CA), which was navigated to the venous side of the fistula. Embolization was carried out with detachable coils (Covidien/ev3) placed from the venous side of the fistula to the arterial side. Onyx-18 (Covidien/ev3) was also used for complete embolization of the fistula (Fig. 2C and D). After the surgery, the patient reported a gradual improvement of his facial pain and complete relief at his 3-month follow-up. At his 6-month follow-up, DSA confirmed no recurrence of the PAVF (Fig. 2E and F), and 3D FIESTA MRI showed shrinkage of the petrosal venous complex and a shift of ACIA away from the right trigeminal nerve (Fig. 1C and F). The patient remained pain-free and neurological exams were unremarkable during 2 years of follow-up.

## 3 Discussion

Compression of the trigeminal nerve entry zone by an aberrant loop of artery or vein accounts for 80% to 90% of TN cases.^[7]^ Other posterior fossa lesions that may result in secondary TN include tumors, aneurysms, vascular malformations, and vertebrobasilar artery ectasia. Regardless of the types of compression, focal demyelination of trigeminal nerve at the REZ is considered to be the primary mechanism. Nevertheless, TN resulting from a PAVF has never been reported in the literature.

PAVF is a rare intracranial vascular lesion characterized by the presence of direct communication of 1 or several arterial feeders into a dilated vein.^[8]^ Patients with PAVF may manifest with different symptoms, including headache, seizure, hemorrhage, and neurological deficits, based mainly on age and the existence of varix.^[8]^ However, no previous cases of PAVF presenting with TN due to the dilated venous pouch and artery have been reported in the literature. In the present case, 2 large venous tributaries of the petrous vein complex and AICA were found close to the nerve, and a mixed venous and arterial compression was suspected to be responsible for the patient's shock-like pain. Normally, the petrosal venous complex drains the brainstem and cerebellum to the superior petrosal sinus and Galen vein. In the present case, the cerebellar PAVF led to venous hypertension of Galen vein, which resulted in retrograde flow of venous blood to the petrous venous complex. The petrous venous complex obviously dilated obviously as a result of the venous hypertension, which produced a mass effect and compressed the trigeminal nerve. MRI showed shrinkage of the petrosal venous complex detaching from the trigeminal nerve after the operation. The AICA, sharing a common trunk with PICA, may also have contributed to the development of TN in this case. Since PICA is the main feeding artery of the PAVF, the common trunk would have an increased blood flow volume, which would also cause high pressure and deformation of the AICA. MRI showed a shift of the AICA away from the trigeminal nerve after the operation, which was consistent with the patient's pain relief.

Mixed venous and arterial compressions of TN as found in our case were also reported in a previous study.^[9]^ However, in the present case, the patient's facial pain was aggravated obviously when he was lying down, indicating that the veins were the main offending vessels as a result of increased intracranial venous pressure. Interestingly, in the present case, the patient's facial pain was not completely resolved after surgery but instead he experienced gradual improvement. We speculated that the patient's delayed pain resolution was due to a gradual recovery of the dilated veins as well as the AICA after the fistula embolization.

The management of PAVF remains an issue.^[10]^ Surgical treatment offers a higher obliteration rate (total obliteration rate of 96.8%, 30 patients). However, surgical treatment would be more difficult when the lesion is small and deep-seated. Endovascular treatment is regarded as a simple and safe option. Another study reported an obliteration rate of 86.5% (32 patients) for endovascular treatment.^[8]^ Coils or liquid embolic agents can be used for the endovascular treatment of PAVF. Coil embolization is a good option for the treatment of PAVF with a small fistula, because the coil is not easily carried away by high-velocity blood flow. In 2006, Luo et al. utilized transarterial Guglielmi detachable coils to treat intracranial high-flow AVFs in single-session embolization and achieved satisfactory results.^[11]^ The liquid embolic agents including NBCA (n-butyl-2-cyanoacrylate) and Onxy have good dispersity, and they can embolize the distal PAVF when the micro-catheter cannot be super-selected to its orifice. Campos et al reported a case of PAVF with good recovery after injection of NBCA.^[12]^ However, there are multiple fistulas or the fistula is very large in some cases, only use of fluid embolization or coils cannot completely embolize the fistula. Combined use of coil and liquid embolic agents can successfully embolized these PAVFs. Youn et al reported that eleven patients who harbored twelve PAVFs were treated with a combination of NBCA and coils and achieved safe and stable occlusions.^[13]^ In the present case, the endovascular approach was chosen because the fistula was easily super-selected by micro-catheter and there was only a PICA solo-feeder. Complete embolization was achieved using a combination of coils and Onyx embolization, and the patient had no recurrence at 6-month follow-up and neurological exams were unremarkable during the 2 years after surgery.

## 4 Conclusions

Endovascular treatment with a combination of coils and Onyx embolization is an effective approach for resolving rarely occurring TN caused by mixed venous and arterial compressions associated with cerebellar PAVF. Additional prospective study of similar rare cases of TN is needed to further confirm long-term clinical outcomes of combination endovascular treatments.

## Author contributions

**Resources:** Shiying Gai.

**Supervision:** Yili Chen.

**Writing – original draft:** Shenghu Wang.

**Writing – review and editing:** Jun Mo, Changjiang Ou.
